# Knowledge, attitude and perception of medical students on COVID-19 vaccines: A study carried out in a Nigerian University

**DOI:** 10.3389/fpubh.2022.942283

**Published:** 2022-09-09

**Authors:** Edidiong Orok, Ekpedeme Ndem, Eunice Daniel

**Affiliations:** ^1^Department of Clinical Pharmacy and Public Health, College of Pharmacy, Afe Babalola University, Ado Ekiti, Nigeria; ^2^Department of Clinical Pharmacy and Biopharmacy, University of Uyo, Uyo, Nigeria

**Keywords:** knowledge, perception, COVID-19 vaccines, medical students, Nigeria

## Abstract

**Objective:**

Vaccine development, distribution, and immunization of large populations have been identified as vital mitigation strategies for curbing the spread of COVID-19. However, vaccine hesitancy is a major barrier to this. The knowledge and perception of COVID-19 vaccines can improve vaccine acceptance. The purpose of this study was to assess medical students' knowledge, attitude, and perception of COVID-19 vaccines.

**Methods:**

This study was a cross-sectional web-based survey conducted among undergraduate students from the faculties of Basic medical sciences and Clinical sciences, University of Uyo, Akwa Ibom State, Nigeria. The students' knowledge was ranked as excellent (>70%), good (50–69%) and poor (<50%) while perception was categorized into positive (>50%), and negative (<50%). The difference in knowledge based on demographics was analyzed using the Independent sample *t*-test. The association between demographics, and students' perception was carried out using the Chi-square and Fisher's Exact tests. Analyses were conducted using SPSS software version 25.

**Results:**

A total of 233 students consented to participate in the study out of which 51.1% were female. Forty-eight students (20.6%) had good knowledge while 41.2% of the participants accepted to take the COVID-19 vaccine. Positive perception was reported among 51.1% (119) of the students.

**Conclusion:**

There was poor knowledge and positive perception among majority of the students. Educational intervention in form of training should be done to improve medical students' knowledge and perception toward COVID-19 vaccination.

## Introduction

Coronaviruses are zoonotic viruses known to cause respiratory infections in humans including the common cold, Middle East Respiratory syndrome coronavirus (MERS-CoV) and severe acute respiratory syndrome coronavirus (SARS-CoV) (1). The outbreak of the new coronavirus, first identified as pneumonia of unknown cause, occurred in Wuhan, China and the genome of this new virus was identified to have a semblance to MERS-CoV and SARS-CoV (1). The World Health Organization (WHO) named this virus 2019-nCoV (Novel Coronavirus 2019) which was later renamed as Severe Acute Respiratory Syndrome Coronavirus-2 (SARS-CoV-2) by the International Committee on Taxonomy of Viruses (2).

Clinical manifestations of SARS-CoV-2 infection are highly variable. Some people with the infection are asymptomatic while others can have mild to moderate symptoms and some other people require intensive care support and in some cases, death especially in older adults (3). People with mild COVID-19 might experience sore throat, cough, diarrhea, headache, high temperature, muscle, or joint pain, fatigue, anosmia (3). Dyspnea is the most common symptom of severe disease and is often accompanied by hypoxemia. Furthermore, progressive respiratory failure develops in many patients with severe COVID-19 soon after the onset of dyspnea and hypoxemia (4).

Currently, some pharmacological treatment options have been approved by the Food and Drug Administration (FDA) to help in the treatment of mild to severe cases of COVID-19 (5). However, preventive treatment in form of vaccines is important to aid in the control of spread and elimination of risk of future occurrences (6).

Vaccines are biological preparations that enhance immunity against diseases and either prevent (prophylactic vaccines) or treat disease (therapeutic vaccines) (7). The Center for Disease Control and Prevention (CDC) defines vaccine as preparation that is used to stimulate the body's immune response against diseases. Vaccines are usually administered through needle injections, but some can be administered by mouth or sprayed into the nose. The act of introducing a vaccine into the body to produce protection from a specific disease is called vaccination (8).

The number of COVID-19 cases reported to the WHO has been growing since the first report of COVID-19 in December 2019. A steady decline in number of new cases was observed since January 2021. However, the CDC has recently seen a rapid and alarming rise in COVID-19 cases and hospitalization rates following the emergence of the delta variant of the virus. New data began to emerge that the Delta variant was more infectious and was easily spread when compared with the other variants of SARS-CoV-2 (9). In Nigeria, 582 new confirmed cases and 11 deaths were reported on the 1st of September, 2021, by the Nigeria Center for Disease Control (NCDC), bringing the total number of confirmed cases to 193,013 and 2,480 deaths (10). Akwa Ibom state is one of the top 20 states with high COVID-19 confirmed cases and deaths along with Lagos, Oyo and Rivers state (11).

Vaccines have proven over the years to be the most effective strategy for preventing infectious diseases as they are more cost-effective than treatment, and reduce morbidity and mortality without long-lasting effects. Preventive and therapeutic vaccines will be of fundamental value as the most obvious way to protect global health (12). Three vaccines have been authorized by the FDA and they include Pfizer-BioNTech^®^, Moderna^®^, Johnson & Johnson^®^, and AztraZeneca COVID-19 vaccines (13).

AztraZeneca was the first COVID-19 vaccine used in Nigeria. On March 2021, Nigeria received nearly 4 million doses of the COVID-19 vaccine, shipped via the COVAX Facility, a partnership between CEPI, UNICEF, and WHO (14). As of 31 August 2021, a total of 4,432,282 vaccine doses have been administered in Nigeria including over 69,000 doses administered in Akwa Ibom state (15). Rapid vaccine development, distribution and immunization of large populations have been identified as vital mitigation strategies for curbing the spread of COVID-19 (16). However, this has been hampered by issues pertaining to vaccine hesitancy.

Vaccine hesitancy occurs when there is a delay in accepting or refusing vaccinations and this has been identified as one of the top 10 threats to world health (17). Issues with vaccine acceptance and hesitancy has been a common problem globally especially in African settings (18). Also, vaccine hesitancy has been reported to be frequent among students and health workers in African countries (19–21). Several factors/reasons have been reported in literature to be associated with vaccine hesitancy particularly in Africa. Reasons such as financial constraints have been reported in Congo (22), Nigeria (23) and Ethiopia (24), although some of the vaccines are administered free in these countries. Similarly, slower infection rates have been purported as a reason for hesitancy in Uganda compared to other countries (25). Furthermore, risk perception, as well as gender differences has been documented as notable reasons for vaccine hesitancy in low income countries (26). Other reasons like unwanted side effects and reduced efficacy has been a frequent stated to be one of the potent reasons for vaccine hesitancy particularly due to reports in European countries of blood clots and other complications associated with AstraZeneca vaccine (27).

There have been studies conducted in different countries to assess COVID-19 vaccine acceptance and hesitancy among medical students. These include COVID-19 vaccine acceptance and hesitancy among medical students in the US (28), COVID-19 vaccine hesitancy among undergraduate medical students in India (29) among others. Studies conducted in Nigeria showed that Nigerians generally were willing to take the COVID-19 vaccines even before it was made available (30). However, in a survey in North Central Nigeria, only 29% would accept COVID-19 vaccines even when 99.5% had good knowledge of it (31). This could be related to past vaccination program that affected participants in the region where inhabitants refused to receive polio vaccinations as a result of Islamic clerics spreading the myth that the vaccine could render women infertile or cause them to contract HIV (32). The polio virus spread more widely across the nation and neighboring nations (33) as a result of northern Nigeria's lower vaccination rates (34). Due to mistrust, similar refusals to take part in polio and tetanus vaccination campaigns have been noted throughout Africa, particularly in Nigeria (35, 36). Furthermore, the knowledge and perception of COVID-19 vaccines can affect vaccine acceptance and hesitancy (37). Also, due to circulating theories in social and traditional media about COVID-19 vaccines inefficiency (38) coupled with history of vaccine boycott (32), good knowledge and perception is paramount in order to make informed decisions regarding COVID-19 vaccination. Medical students can help spread awareness during times of public health crisis. Medical students have been active in public health campaigns during earlier viral epidemics, such as the human immunodeficiency virus, influenza, severe acute respiratory syndrome, and Ebola (39). Medical students may act as role models for the public to embrace COVID-19 preventative health practices since they are seen as having a greater degree of health literacy. Medical students have contributed to the public's access to health information during the COVID-19 pandemic, mostly by using social media and other sources (40). Furthermore, medical students, as future healthcare providers, should have adequate knowledge on COVID-19 vaccines since they may be saddled with the responsibility to provide vaccine recommendations and counseling to vaccine-hesitant patients. Comprehensive evaluations of medical students' knowledge, attitude, and perception are necessary to further improve their potentials in educating the public. Particularly, this might offer essential data to stakeholders for identifying field gaps and developing initiatives to better motivate communities to adhere to health standards. Additionally, medical institutions may use this information to enhance their curricula to better prepare students for future epidemics. To the best of our knowledge, no study has been conducted in Nigeria to analyse medical students' knowledge, attitudes, and perceptions of COVID-19 vaccines. The purpose of this study was to assess medical students' knowledge, attitudes, and perceptions of COVID-19 vaccines in clinical and fundamental medical sciences.

## Methods

This study was a cross-sectional web-based survey conducted among undergraduate students in the faculties of Basic medical sciences and Clinical sciences in the University of Uyo, Akwa Ibom State, Nigeria, from May to June, 2021. Faculty of Basic medical sciences comprises of three departments namely: Department of Human Anatomy, Department of Medical Physiology and Department of Biochemistry. Faculty of Clinical sciences comprises of the Department of Medicine and surgery. The study population consisted of male and female undergraduate students of all levels in the departments of Medicine and surgery, Human Anatomy, Biochemistry and Medical Physiology in the University of Uyo.

The sample size was calculated using Yamane formula (41).


(1) $$\begin{array}{r} n=\frac{N}{1-N\left(e^{2}\right)}\; \end{array}$$

where n is the sample size, *N* is population size and e is the level of precision.

Population size (*N*) = 2,077 students which was gotten by summing up the total number of students in each classes; *e* = 0.05.

By applying the formula,

*n* = 2,077/1-[2,077(0.05)^2^] = 335 students

The questionnaire was a semi-structured questionnaire developed after a thorough literature search using google forms. It was divided into four sections. The first section briefly explained the purpose of the research. The second section documented participants' demographics which included the age, gender, marital status, department and year of study. The third section measured students' general knowledge about COVID-19 vaccines. The fourth section assessed participants' attitude and perception toward COVID-19 vaccines. Perception were assessed by 5 point Likert scale questions allocated. Student perception was determined by


$$\begin{array}{l} \left(\frac{Student\;perception\;score}{Total\;obtainable\;perception\;score}\right)\times 100\% \end{array}$$

The questionnaire was reviewed by experts, who were lecturers of the department of Clinical Pharmacy and Biopharmacy, University of Uyo, and revised based on their comments. The questionnaire was pretested among 20 students who were excluded from the main study. The reliability of the questionnaire was determined using the Cronbach alpha reliability test and a score of 0.75 was obtained. The link for the questionnaire was exclusively shared with undergraduate medical students both individually and through their social media groups (WhatsApp groups) with the assistance of their respective class representatives. The online survey took about 5 min to complete and was designed to ensure duplicate entries were avoided. The questionnaires ensured exclusivity to only medical students by including only medical courses and instructions stating that the study is strictly for medical students.

Data collected were coded and all the analysis was done using the Statistical Package for Social Sciences version 25 (SPSS 25.0) software. The students' socio-demographics, perception and attitude about COVID-19 vaccine were summarized using frequency counts and percentages. Knowledge scores of the students on COVID-19 vaccine was summarized using mean ± standard deviation and their overall knowledge was categorized into excellent (>70%), Good (50–69%), and poor knowledge (<50%). The students' perception was categorized into positive (>50%) and negative (<50%). The difference in knowledge scores on COVID-19 vaccines based on gender and marital status was analyzed using the Independent sample *t*-test. The association between demographics, source of information and students' perception of COVID-19 vaccines was carried out using the Pearson's Chi-square and Fisher's Exact test. All significant differences was set at *p* < 0.05

## Results

A total of 233 students participated in the study. Most of the respondents (91.4%) were between the ages of 18 and 27 years. One hundred and fourteen (48.9%) students were males and 42.9% (100) of the students were from the department of Medicine and Surgery while majority of the students were in their pre-clinical year (154, 66.1%). The summary of demographic characteristics is presented in Table 1.

**Table 1:** Demographics of the study participants.

**Demographics**		**Frequency (%)**
Gender	Male	114 (48.9)
	Female	119 (51.1)
Age	<18 years	16 (6.9)
	18–27 Years	213 (91.4)
	Above 27 years	3 (1.3)
Marital status	Single	228 (97.9)
	Married	5 (2.1)
Departments	Anatomy	25 (10.7)
	Biochemistry	65 (27.9)
	Medicine and surgery	100 (42.9)
	Physiology	43 (18.5)
Years of study	100–300 level (Pre-Clinical)	154 (66.1)
	400–600 level (clinical)	79 (33.9)

94.8% (220) knew that COVID-19 is real and deadly. Most of the participants (56.6%) failed to identify correct doses and recommended dosing schedule for OXFORD/AstraZeneca vaccine. Overall, a small population of respondents (2.6%) had excellent knowledge, while 76.8% had poor knowledge. The mean Knowledge score was 4.30 (*SD*: 2.746) (Table 2). Mean knowledge score ± *SD* for male and female participants were 4.16 ± 2.762 and 4.46 ± 2.732, respectively. Nearly half of the participants (43.27%) did not know about types of COVID-19 vaccines while 25% knew about mRNA vaccines and viral vector-based COVID-19 vaccines. Twenty seven participants (11.59%) respondents were not aware that vaccines were available in Nigeria and 102 (43.78%) were not sure which of the vaccines was in use. Sources of information of the participants on COVID-19 pandemic and vaccines identified by the students included print/electronic media (2, 0.8%), social media (140, 60%), as well as friends and colleagues (6, 2.4%) (Table 2). Ninety-six (41.2%) participants accepted to take the vaccines while one hundred and nine (46.8%) participants agreed to encourage others to take the vaccines.

**Table 2:** Knowledge statements of study participants.

**S/N**	**Statement**	**Responses (%)**	
		**Correct**	**Incorrect**
1	COVID-19 is real and can get severe and cause death	221 (94.8)	12 (5.2)
2	Two doses given 8–12 weeks apart is the recommended dosing schedule for OXFORD/AstraZeneca COVID-19 vaccine	101 (43.3)	132 (56.6)
3	Adults >18 years, adults with co-morbidities, healthcare professionals with frequent patient contact and those currently diagnosed with COVID-19, should take COVID-19 vaccine	68 (29.2)	165 (70.8)
4	The number of doses for the AstraZeneca vaccine is 2 doses and it should be taken through the intramuscular route	73 (31.3)	160 (68.7)
5	I know how COVID-19 vaccines work	Yes	96 (41.2)
		No	137 (58.8)
	Mean Knowledge score ±*SD* (Range): 4.30 ±-2.746 (0–14) Total obtainable score: 14		
	Knowledge categories	Excellent knowledge (>70%)	6 (2.6)
		Good knowledge (50–69 %)	48 (20.6)
		Poor knowledge (<50%)	179 (76.8)
	Source of information by the participants		
	Information source	Frequency (%)	
	Print/Electronic media	2 (0.8)	
	Friends and colleagues	6 (2.4)	
	International and health organizations	7(3.2)	
	Medical Journals	8 (3.4)	
	Health professionals	70 (30)	
	Social media	140 (60)	

Ninety-one students (38.89%) stated that they will rely only on doctor's recommendation to accept the vaccines while others reported that they would accept the vaccines out of fear of contracting the virus and getting ill (7.41%) or spreading the virus to family and friends (23.15%). The major reason for rejection of the vaccines was lack of trust in the vaccine's safety and efficacy (36.11%) 8.33% (6) of respondents also revealed that they lacked trust in the vaccine source and in the government. Most of the participants (119, 51.1%) had positive perception of COVID-19 vaccines. 41.6% agreed that COVID-19 vaccination is an effective way to control and prevent COVID-19.30.4% felt COVID-19 vaccines are safe while 70.4% of the respondents expressed concerns about the side effects of the vaccines. A good number expressed confidence that the benefits outweigh any possible side effects (21% strongly agreed and 27.5% agreed) (Table 3).

**Table 3:** Perception of study participants on COVID-19 vaccine.

**S/N**	**Statement**	**Responses (%)**				
		**SA**	**A**	**UN**	**D**	**SD**
1	COVID-19 vaccine is an effective way to control and prevent COVID-19	79 (33.9)	97 (41.6)	43 (18.5)	10 (4.3)	4 (1.7)
2	COVID-19 vaccines should be made compulsory for the general population	45 (19.3)	45 (19.3)	58 (24.9)	52 (22.3)	33 (14.2)
3	COVID-19 vaccines are unsafe	12 (5.2)	25 (10.7)	125 (53.6)	49 (21.0)	22 (9.4)
4	The vaccines are not necessary, the immune system is enough	7 (3.0)	24 (10.3)	78 (33.5)	75 (32.2)	49 (21.0)
5	I am afraid that the vaccines might have unwanted side effects	69 (29.6)	95 (40.8)	52 (22.3)	12 (5.2)	5 (2.1)
6	The benefit of the vaccines outweighs possible side effects	49 (21.0)	64 (27.5)	92 (39.5)	25 (10.7)	3 (1.3)
7	I believe the vaccines would have future medical implications	30 (12.9)	41 (17.6)	134 (57.5)	17 (7.3)	11 (4.7)
	**Positive perception (>50%)** 119 (51.1)					
	**Negative perception (<50%)** 114 (48.9)					
	**Attitude Statements**	**Responses (%)**				
		**Yes**		**No**	**I don't know**	
	I will accept the COVID-19 vaccine	**96 (41.2)**		**73 (31.3)**	**64 (27.5)**	
	I will encourage others to accept COVID-19 vaccine	**109 (46.8)**		**46 (19.7)**	**78 (33.5)**	

SA, Strongly Agree=5; A, Agree=4; UN, Undecided=3; D, Disagree=2, SD, Strongly Disagree=1.

There was no significant difference in knowledge scores between both gender (*p* = 0.405) as well as based on study levels of the participants (*p* = 0.089) (Table 4). There was no significant association between source of information and perception of the students (*p* = 0.567).

**Table 4:** Association between knowledge scores, perception and demographics of participants.

**Item**	**Demographics**									**Source of information**						
	**Gender**		***P***	**Marital status**		***P***	**Study levels**		***P***	**SM**	**HP**	**MJ**	**IH**	**FC**	**PM**	***P***
	**Male**	**Female**		**Single**	**Married**		**100–300**	**400–600**								
Mean knowledge score ±*SD*	4.16± 2.762	4.46 ±-2.732	0.405^a^	4.23 ±-2.766	4.50 ± 3.109	0.845^a^	4.30 ±-2.350	4.50 ± 3.091	0.089	
Positive perception	63	56	0.962^b^	117	2	0.991^c^	71	44	0.567	60	40	3	5	1	1	0.567^c^
Negative perception	60	54		112	2		83	35		80	20	5	2	5	1	

^a^Independent sample t-test.

^b^Pearson Chi-square test.

^c^Fisher's Exact test.

SM, Social Media; HP, Health professionals; MJ, Medical Journals; IH, International&other health organizations; FC, Friends &colleagues; PM, Print/Electronic Media.

There was also no significant association between perception and gender (*p* = 0.962) and between perception and marital status (*p* = 0.991) (Table 4).

## Discussion

Majority of the study participants agreed that COVID-19 is real, can get severe and cause death.

During pandemics such as COVID-19, healthcare systems are put under great pressure, and paucity of healthcare providers can lead to the involvement of less experienced healthcare providers such as medical students (42). This is because medical students and health workers show good knowledge of COVID-19 disease and high level of performance in preventive behaviors (43, 44).

Most of the participants incorrectly answered questions related to doses, dosing schedule, route of administration, category of people that should take the vaccines, types of COVID-19 vaccines, the COVID-19 vaccines available and used in Nigeria and more than half had poor understanding of how the vaccines work. The results obtained showed that most medical students had poor knowledge of COVID-19 vaccines. This is dissimilar to similar studies where medical students showed good knowledge (43, 44). Educational intervention in form of regular training should be done to improve knowledge of medical students on COVID-19 vaccines.

Social media was reported as one of the major sources of information by the participants. This is in accordance with a similar study among Turkish university healthcare students where social media was a major information source for learning about the influenza pandemic (45), but in slight contrast when examining studies on less covered subjects such as the Zika virus epidemic where news outlets seemed to be the main source of information (46). There is possibility that turning to social media for information on vaccines played a role in development of COVID-19 vaccine hesitancy because of its potential of disseminating misleading health information (47). This should alert policy makers to the importance of social media in disseminating information to the public especially in cases of pandemics. Students should also be properly guided to proper sources of information and also be equipped with medical knowledge, proper attitude, and good precautionary measures. More frequent utilization of social media by medical schools to spread knowledge should be placed to implement dissemination of information involving public health emergencies.

Doctor's recommendation was the major factor influencing vaccine acceptance. This is similar to findings reported in a US-based study where it was documented that participants were most likely to accept COVID-19 vaccines if they thought that their healthcare provider would recommend them (48). Healthcare providers are seen as one of the most trusted source for information on COVID-19 vaccines (49).

Lack of trust in the vaccine's safety and efficacy was the major reason for vaccine hesitancy. This is similar to a nationwide survey carried out in India where concerns regarding safety of COVID-19 vaccine and its efficacy were the most common reasons cited by those hesitant to take the vaccine (29). Many other studies have documented concerns regarding COVID-19 vaccine adverse events as a possible reason for hesitancy among university students and general population (29). These reasons are validated in a recent study that showed that increasing efficacy as well as decreasing incidence of adverse effects is associated with a higher probability of accepting a vaccine (50). Availability of effective vaccines with lesser side effects can improve COVID-19 vaccine acceptance. Although the efficacy of vaccines against the spread of COVID-19 has not necessarily translated to a reduction in vaccine hesitancy (51, 52).

Risk perception was another factor that positively influenced participants' decision to accept the vaccines as 7.41% were afraid of contracting the virus and getting ill and 23.15% were afraid of contracting the virus and spreading it to family and friends. This is in tandem to findings from studies in the UK and Australia where it was reported that an increased perceived risk of developing severe disease was identified as significant facilitating factor toward COVID-19 vaccine acceptance among adults (48, 53, 54). Presence of risk perception among students regarding COVID-19 disease has also previously been shown to be associated with lesser hesitancy (29).

Reasons for rejecting COVID-19 vaccines included inadequate information regarding the adverse effects of the vaccine, lack of trust in the vaccine source and in the government, and belief that the vaccines would have future medical implications. These challenges are not new as a similar result was obtained from a study carried out among medical students in the U.S. where vaccine hesitancy was observed among 23% of the students due to concerns about serious vaccine side effects and lack of trust in the information received from public health experts (28). Some of the students lacked trust in the government and made comments such as politicization of the vaccine and need for transparency. Participants in a U.S.-based study also showed concerns about the speed of vaccine development potentially impacting vaccine safety and about possible congenital defects in babies born to mothers who received the new vaccine (28).

Providing scientifically sound information about the benefits of vaccination (55) or modifying information based on culture can effectively educate the populace (56). The impact of education on attitude can be seen in studies such that interventions channeled toward addressing the fear of needles and injection may play an important role in curtailing vaccine reluctance (57).

There was an overall positive attitude toward vaccination among the healthcare students. This is similar to findings from a study from south eastern Nigeria where the population of Nigeria adults showed a positive attitude toward COVID-19 vaccines. However, it is dissimilar to a study in Jordan where most of the public were negative about COVID-19 vaccines. This could be due to a low perceived risk of the disease (58). In this study, the positive attitude is important as it can influence the future behavior of the students as medical professionals, and parents who can help improve the future perspective of the society at large on vaccination.

Though a good number of the participants agreed that COVID-19 vaccination was an effective way to control and prevent COVID-19, 10% regarded the vaccine as unnecessary, opining that the immune system was enough. A similar anti-vaccine attitude was observed among U.S. adults where 10.8% did not intend to be vaccinated owing to vaccine-specific concerns, a need for more information, anti-vaccine beliefs and a lack of trust (59). A previous study had also shown that some Africans feel immune to COVID-19 (38).

Overall, 51.1% positive perception was observed among participants and 48.9% negative perception. This differs slightly from a previous study that showed that the perception of Nigerians toward COVID-19 vaccine trial was 61% positive and 39% poor (60).This study is not without limitations. The study did not mention the side effects of the AstraZeneca vaccine and other COVID-19 vaccines. Also, there was no provision to assess if participants preferred other COVID-19 vaccines such as Moderna^®^, and Johnson & Johnson^®^. These may have contributed to the attitude and perception of the students toward COVID-19 vaccines.

## Conclusion

This study shows poor knowledge, positive attitude and positive perception among majority of the medical students. Educational intervention in form of regular training of medical students is warranted in order to improve their knowledge and perception of COVID-19 vaccines

## Data availability statement

The raw data supporting the conclusions of this article will be made available by the authors, without undue reservation.

## Ethics statement

The studies involving human participants were reviewed and approved by University of Uyo/UUTH Research Ethics Committee. The patients/participants provided their written informed consent to participate in this study.

## Author contributions

EN: conceptualization and writing—review and editing. ED: data curation, formal analysis, writing—original draft, and writing—review and editing. EO: conceptualization, methodology, data curation, formal analysis, visualization, writing—original draft, and writing—review and editing. All authors contributed to the article and approved the submitted version.

## Conflict of interest

The authors declare that the research was conducted in the absence of any commercial or financial relationships that could be construed as a potential conflict of interest

## Publisher's note

All claims expressed in this article are solely those of the authors and do not necessarily represent those of their affiliated organizations, or those of the publisher, the editors and the reviewers. Any product that may be evaluated in this article, or claim that may be made by its manufacturer, is not guaranteed or endorsed by the publisher.
